# Stressful situations: Molecular insights on mitochondrial quality control pathways

**DOI:** 10.1016/j.jbc.2025.110483

**Published:** 2025-07-16

**Authors:** Sabrina Romanelli, Jean-François Trempe

**Affiliations:** 1 Department of Pharmacology and Therapeutics, McGill University, Montreal, Quebec, Canada; 2 Centre de Recherche en Biologie Structurale, McGill University, Montreal, Québec, Canada

**Keywords:** mitochondria, import, stress response, unfolded protein response, PINK1, Parkin, DELE1, mitophagy, ubiquitin

## Abstract

Mitochondrial quality control has emerged as an important area of research over the past decade, with more than 2000 publications exploring the molecular pathways that regulate it. Mitochondria are essential for energy production and various cellular functions but are highly susceptible to damage from stressors such as protein misfolding, reactive oxygen species, and chemicals that disrupt the electron transport chain. If left unresolved, mitochondrial dysfunction can lead to health complications, including neurodegenerative disorders, cardiovascular diseases, and cancer. To maintain cellular health, cells evolved quality control pathways to remove damaged mitochondrial components. This review focuses on three key quality control responses: the PTEN-induced kinase 1*–*Parkin pathway, the DELE1*–*HRI pathway, and the mitochondrial unfolded protein response. While these pathways have distinct functions, there is ongoing debate about how they overlap and which responds first in different contexts. In this review, we discuss the physiological and structural mechanisms behind each pathway, explore how they interconnect, and highlight their differences and relevance to disease. By summarizing this information in a single review, we aim to enhance the molecular understanding of mitochondrial quality control, which can help highlight avenues for novel therapeutics for diseases associated to dysfunctional mitochondria.

Mitochondria are complex double-membrane bound organelles found in nearly all cells. They are commonly known for their role in energy production through ATP generation by oxidation phosphorylation, needed for cellular processes (1). These organelles also play an important role in calcium homeostasis, innate immunity, heat production, and regulating programmed cell death (2, 3, 4, 5). To mediate these roles, human mitochondria have around 1500 different proteins, with 99% of them nuclear-encoded (6). These proteins need to be imported as unfolded precursors to the organelle, using different mitochondrial import pathways (6, 7). This process is critical, if proteins do not fold properly or aggregate, it can lead to proteotoxic stress in the mitochondria, resulting in its dysfunction (8). In addition to protein misfolding, mitochondria are subjected to other stressors. Due to their role in energy production, mitochondria are exposed to high amounts of reactive oxygen species (ROS), which can cause damage to mitochondrial proteins, membranes, and DNA (8, 9, 10). Furthermore, exposure to environmental toxins, nutrient deprivation and mutations in mitochondrial DNA negatively impact mitochondrial function (9). These mitochondrial stressors are immensely detrimental because mitochondria are vital to many essential cellular functions and their dysfunction has been linked to neurological disorders such as Parkinson’s disease, Alzheimer’s disease, and ataxia, as well as other diseases, such as diabetes and cancer (7, 9, 11, 12, 13). Therefore, organisms have evolved cellular pathways to counteract the impact of mitochondrial stressors and maintain cellular function and health. Different pathways are present in cells that are responsible for safeguarding the mitochondria, from whole organelle mitophagy to selective turnover of mitochondrial subcompartments. Here, we will critically review three distinct pathways responsible for the turnover of mitochondria upon induction of mitochondrial stress in humans, namely the mitochondrial unfolded protein response (UPR^mt^), the DELE1-HRI integrated stress response and the PTEN-induced kinase 1 (PINK1)–Parkin quality control pathway (Table 1). We will describe the pathways, with an emphasis on their molecular and structural mechanisms—this level of understanding is essential to understand how the pathway components “sense” damage and how mutations in these pathways cause diseases. We will also comment on how each of the pathways work independently of one another, while also addressing new literature emerging that there might be crosstalk between the different damage responses. Lastly, we will highlight the importance of understanding these pathways, connecting them to relevant diseases and stressing their therapeutic importance.

**Table 1:** Three key stress response pathways in mammalian cells and the way in which each pathway is activated and repairs damage

Mitochondrial quality control pathways	Cellular/molecular stressors that trigger the activation of the response	Pathways by which damage is removed by the stress response
*Mitochondrial unfolded protein response (UPR*^*mt*^*)*	• Reactive oxygen species in cytosol (29) • Precursor proteins/misfolded proteins in cytosol (29)	• Upregulation of mitochondrial chaperones (*e.g.*, HSPE1, HSPD1, mtHsp70) and proteases (*e.g.*, LONP1) (19, 32)
*DELE1–HRI pathway*	• Inhibition of mitochondrial HSP90 (47, 50) • Mitochondrial membrane depolarization (47) • TIM23 subunit knockdown in the TIM complex (50) • Inhibition of the PAM complex (47, 48, 50)	Mild damage: • Slowdown of global translation (40, 43) • Expression of specific genes needed for cellular repair (*e.g.*, ATF4 and CHOP) (46, 47) Severe damage: • Mitophagy (115)
*PINK1–Parkin Pathway*	Distinct stressors: • Mitochondrial membrane depolarization with protonophores (156) • Inhibition of electron transport chain (63, 76, 78, 157) • mtDNA mutations (79) • Small mitochondrial ARF (77) • Unfolded protein accumulation and inhibition of mitochondrial HSP90 (18, 109)	Mild damage: • Formation of mitochondrial-derived vesicles (MDVs) (90) Severe damage: • Mitophagy (86, 87)

PAM, presequence-associated motor; TIM, translocase of the inner membrane.

## Import of proteins to the mitochondria and its relationship to dysfunctional mitochondria

Most mitochondrial proteins are encoded by nuclear genes, synthesized in the cytosol, and imported into the mitochondria (14). This import process starts in the cytosol, where unfolded protein precursors are translated and bound by chaperones to prevent aggregation and maintain an unfolded state before entering the mitochondria (15). Depending on their mitochondrial targeting signal (MTS), the unfolded precursors can be localized to one of the four subcompartments of the mitochondria, such as the outer mitochondrial membrane (OMM), the inner membrane space (IMS), the inner mitochondrial membrane (IMM) or the matrix (6). The majority of precursor proteins (>90%) will enter the mitochondria through the translocase of the outer mitochondrial membrane (TOM) complex, a multimeric protein channel in the OMM, and will then be imported to their respective sub-compartment *via* different pathways (7). The import of proteins into the mitochondria is thus a complex and highly regulated process which acts as a barometer of mitochondrial dysfunction. For instance, exposure to high levels of ROS can disrupt the protein import process (16). When exposed to paraquat, a NADH:ubiquinone oxidoreductase inhibitor that triggers superoxide generation, the respiratory chain is disrupted, leading to increased ROS production (16). Elevated ROS levels hinder mitochondrial protein import efficiency, resulting in an accumulation of unfolded protein precursors in the cytosol (16). This buildup of misfolded proteins adds further stress to the mitochondria by overwhelming the chaperones in the mitochondrial matrix. Furthermore, other studies have highlighted that impairing the ability of mitochondrial chaperones to assist in protein folding or chemically disrupting the mitochondrial membrane potential can further compromise protein import (17, 18). These stressors lead to proteotoxicity and ultimately mitochondrial dysfunction. Mitochondrial protein precursors are also susceptible to age-related damaged. As organisms age, protein import into the mitochondria becomes increasingly vulnerable, with a rise in chaperone defects, ROS production, and the accumulation of misfolded proteins (10). Therefore, organisms must develop effective mechanisms to manage this damage. In the next sections, we will describe the different pathways present in humans that act as surveillance quality control systems (summarized in Table 1).

## Repairing the damaged mitochondria–stress response pathways

### Mitochondrial unfolded protein response

**Figure 1:**
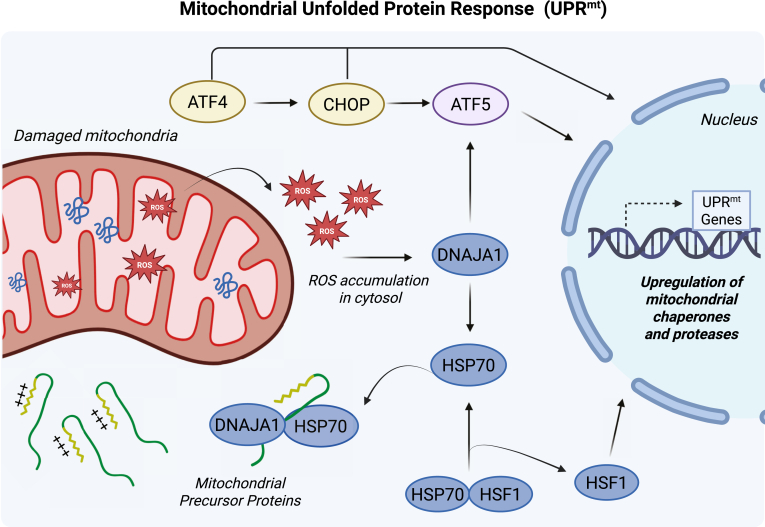
**Summary of the mitochondrial unfolded protein response in mammalian cells.** The accumulation of mitochondrial precursor proteins and reactive oxygen species (ROS) in the cytosol triggers the activation of the UPR^mt^. Various transcription factors play essential roles in initiating this response. When there is mitochondrial stress, ROS is released into the cytosol to activate the cochaperone DNAJA1. DNAJA1 can then recruit HSP70 to the damaged mitochondria to alleviate stress. As HSP70 dissociates from heat shock factor 1 (HSF1), HSF1 translocates to the nucleus, leading to the upregulation of UPR^mt^ genes, causing the expression of mitochondrial chaperones and proteases. This DNAJA1–HSF1 axis can also regulate ATF5, another transcription factor that activates the UPR^mt^. Additionally, ATF4 and CHOP can collaborate with ATF5 or act independently to promote the UPR^mt^. The upregulated chaperones and proteases work to repair the damaged mitochondrial.

To mitigate proteotoxic stress, mitochondria have evolved a stress response that relay their internal proteotoxic stress to the cytosol and nucleus. This response, called the UPR^mt^, initiates a cascade of transcription factors that upregulate stress-response genes, namely mitochondrial chaperones and proteases (19, 20, 21) (Fig. 1). Upregulation of chaperones aid in refolding misfolded proteins, while proteases help remove any remaining damaged proteins (22). Thus, the UPR^mt^ specifically repairs the damaged mitochondria compartment, rather than cause the degradation of the entire organelle (23). The UPR^mt^ is vital for maintaining mitochondrial health, as mitochondria do not encode stress response proteins in their genome (24).

The UPR^mt^ was first discovered in mammalian cells, where inducing unfolded proteins in the mitochondrial matrix, through an ornithine transcarbamylase mutant deletion (ΔOTC) or mitochondrial DNA deletion, triggered an increase in stress-response proteins (19, 20). The mechanism underlying this stress response pathway has been most extensively characterized in *Caenorhabditis elegans* (21, 25, 26, 27, 28). Only recently have studies begun to elucidate the underlying mechanism in humans. A pivotal study by Christian Münch's group found that the accumulation of mitochondrial precursor proteins and elevated levels of ROS in the cytosol act as key signals that work together to initiate the UPR^mt^ (29). To investigate this stress-response pathway, Münch's group used the chemical gamitrinib triphenylphosphonium (GTPP) to induce mitochondrial protein misfolding (29). Gamitrinib is an HSP90 inhibitor, with the triphenylphosphonium moiety targeting it to mitochondria, where it inhibits the HSP90-like chaperone TRAP1/HSP75, involved in folding protein precursors in the matrix (30). After GTPP treatment, precursor proteins accumulated in the mitochondrial matrix and the cytosol, leading to mitochondrial stress measured by the accumulation of insoluble proteins (29). This stress simultaneously triggered an increase in mitochondrial ROS, which diffused into the cytosol over time. Inhibiting either ROS diffusion with n-acetyl-cysteine or reducing precursor protein accumulation in the cytosol by inhibiting translation, reduced UPR^mt^ activation, emphasizing the role of these stressors in triggering the response (29). Using proteomics, the DNAJA1–HSF1 axis was identified as being necessary for the activation of the human UPR^mt^ (Fig. 1). According to their model, upon mitochondrial stress, ROS is released in the cytosol, which oxidizes the cytosolic HSP40 cochaperone DNAJA1 on Cys149/150. Oxidized DNAJA1 then recruits cytosolic HSP70 to the OMM where it interacts with unfolded mitochondrial precursors. This triggers activation of the transcription factor HSF1, which is bound to HSP70 under basal conditions but dissociates when HSP70 engages with a substrate (31, 32). Active HSF1 then translocates to the nucleus, where it triggers the expression of stress-response genes (also known as UPR^mt^ genes), such as mitochondrial chaperones (*e.g.*, HSPE1, HSPD1, mtHsp70) and proteases (*e.g.*, LONP1) (29, 33). However, the structural basis for this ROS-induced switch remains to be uncovered. While the interaction between HSP40’s J-domain and HSP70 members is well characterized (34), it is unclear how oxidation of Cys149/150, located in a zinc-finger like region downstream of the J-domain, enhances this interaction. These questions will be critical to dissect further these mechanisms and elaborate therapies. This study nonetheless uncovered signaling molecules that initiate the human UPR^mt^ and emphasizes that the UPR^mt^ can be activated even when the mitochondrial membrane potential remains intact, indicating that depolarization is not necessary for the activation of the UPR^mt^ (29, 35).

Previous studies in *C. elegans* have identified several transcription factors that play a role in initiating UPR^mt^ activation (11). This involvement of multiple transcription factors to activate the pathway appears to extend to mammalian cells as well. For example, the transcription factor ATF5, the mammalian homolog of ATFS-1 in *C. elegans*, has also been shown to activate the UPR^mt^ in humans (35). ATF5 contains both an MTS and a nuclear localization signal, which are essential for its function (35). Under homeostatic conditions, ATFS1-1/ATF5 is imported into mitochondria, where it is degraded by the LONP1 protease (36). Upon mitochondrial damage that reduces import efficiency, ATF5 is no longer imported and accumulates in the cytosol, allowing it to translocate to the nucleus (35). Once at the nucleus, ATF5 will increase the expression of UPR^mt^-related genes to help mitigate the damage. Interestingly, it was found that transcription of ATF5 is regulated by the DNAJA1–HSF1 axis, suggesting crosstalk between these two distinct pathways (29). Other transcription factors in humans, such as CHOP and ATF4, have also been associated with regulating the expression of UPR^mt^ genes and are regulated by import efficiency (23, 37, 38). ATF4, ATF5, and CHOP thus all play important roles in activating the human UPR^mt^ (23). In fact, a recent transcriptome analysis examining UPR^mt^ gene expression revealed that UPR^mt^ genes require one, two, or all three of these transcription factors for their upregulation (23). This aligns with prior studies showing that ATF4 can induce CHOP expression to enhance stress-response gene expression and that both CHOP and ATF4 can facilitate ATF5’s accumulation in the nucleus (38, 39). Hence, multiple transcription factors activate the UPR^mt^ in humans, with more yet to be discovered. Indeed, transcriptome analysis of different UPR^mt^ genes upregulated after mitochondrial stress, showed only 50% of the genes were under the regulatory control of either CHOP, ATF4, or ATF5, suggesting other transcription factors are important for this response (23). Furthermore, the tissue specificity of the various transcription factors associated with the UPR^mt^ (CHOP, ATF5, ATF4, HSF1) has not been well-studied (23). This would be a crucial area to examine as tissue specificity of these factors can be important for creating targeted therapeutic approaches.

### DELE1-HRI integrated stress response

The ISR is another pathway that alleviates mitochondrial stress through reprogramming gene expression (40). This response is an evolutionarily conserved mechanism that aids in maintaining cellular health when exposed to various environmental stressors. The ISR can be triggered by one of four different kinases, which phosphorylate the eukaryotic translation initiation factor (eIF) 2α (41). Phosphorylation of eIF2α disrupts the formation of the eIF2α ternary complex, which is needed for initiating mRNA translation during protein synthesis (40, 42). As a result, phosphorylation of eIF2α activates the ISR, which protects cells through two key mechanisms. The first mechanism slows global translation, lessening the burden on the mitochondrial proteome during stress (40, 43). Simultaneously, the second mechanism activates the expression of specific genes, such as ATF4, which help the cell repair itself by upregulating genes involved in the resistance to oxidative stress and amino acid metabolism (43, 44, 45). However, up until recently, the mechanisms underlying the ISR were unknown.

**Figure 2:**
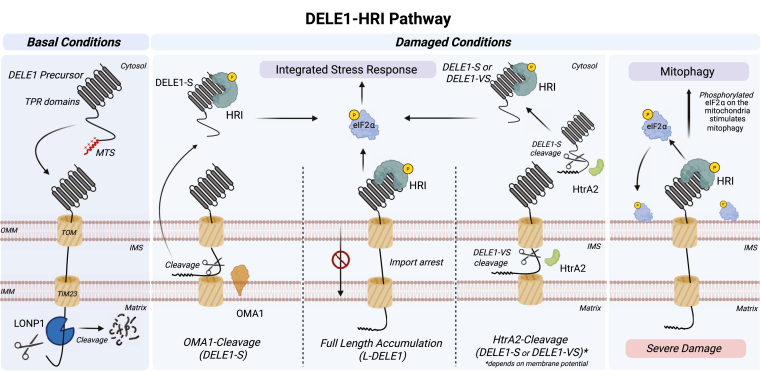
**DELE1–HRI pathway under basal and damaged conditions.** Under basal conditions, DELE1 (made up of seven tetratricopeptide (TPR) domains and a mitochondrial targeting signal (MTS)) is imported into the mitochondria through the translocase of the outer membrane (TOM) and translocase of the inner membrane (TIM23) complex. DELE1 is then rapidly degraded by the AAA+ protease LONP1. Under various stressors the DELE1-HRI integrated stress response is activated, through binding of DELE1 to the kinase HRI in the cytosol. This process can happen three ways. 1, full-length DELE1 is cleaved by the IMM-protease OMA1, resulting in the C-terminal fragment DELE1-S. DELE1-S then binds HRI to activate the kinase. 2, full-length DELE1 (L-DELE1) can bind to HRI as well to activate the kinase. 3, another protease HtrA2 can cleave DELE1. HtrA2 will change location depending on membrane polarization, under depolarized conditions, HtrA2 will cleave DELE1 in the cytosol into DELE1-S and under polarized conditions, HtrA2 will cleave DELE1 in the inner membrane space (IMS) into DELE1-VS. These different DELE1 forms (DELE1-S, DELE1-VS, and L-DELE1) bind HRI, leading to its activation and phosphorylation of eIF2α to activate the integrated stress response. Under conditions of severe damage, phosphorylated eIF2α accumulations on the mitochondria, initiating mitophagy. IMM, inner mitochondrial membrane; eIF, eukaryotic translation initiation factor.

By using genome-wide screenings, two groups in 2020 identified the DELE1–HRI axis as the key mechanism that activates the human ISR (46, 47). These studies suggested that DELE1, a mitochondrial inner membrane protein, binds and activates the kinase HRI in the cytosol, which then phosphorylates eIF2α to trigger the ISR through induction of the ATF4 and CHOP transcription factors (Fig. 2) (46, 47). Under normal conditions, DELE1 is imported into the mitochondria *via* the presequence import pathway (48). DELE1 consists of seven tetratricopeptide repeat (TPR) domains in the C terminus and an N-terminal MTS (43, 47, 48). The MTS of DELE1 allows the protein to be imported into the mitochondrial matrix through the TOM and presequence translocase of the inner membrane (TIM23) complexes. Once in the mitochondrial matrix, DELE1 is cleaved by the mitochondrial processing peptidase and rapidly degraded by the AAA+ protease LONP1 (49). However, when exposed to different mitochondrial stressors such as depolarization or GTPP treatment (Table 1), DELE1 is no longer degraded by LONP1 and accumulates in the cytosol (Fig. 2) (47, 48, 50). One of the first studies to explore how DELE1 localizes to the cytosol demonstrated that, under depolarization, the IMS protease OMA1 cleaves full-length DELE1 at His142 (46, 50). This cleavage of DELE1 generates a smaller C-terminal fragment, named DELE1-S, which contains the TPR domains of the protein and activates HRI in the cytosol (46, 47). The structural basis for DELE1 activation of HRI remains to be elucidated; however, it is likely through interacting with the TPR domains of DELE1 (43, 47). Deletion mutants in the TPR-containing region of DELE1 impact HRI activation, supporting the importance of these domains for DELE1’s interaction with HRI (47).

Stephen E. Girardin’s group recently discovered that the serine protease HtrA2 is also capable of cleaving DELE1 during import stress, generating two distinct forms depending on the mitochondrial membrane potential (50) (Fig. 2). The location of this protease will change depending on the stressor (51). In depolarized mitochondria, HtrA2 is localized in the cytosol and cleaves DELE1 to generate the same DELE1-S fragment as OMA1 (46, 50). In contrast, under nondepolarizing stress, HtrA2 is located in the IMS and will cleave DELE1 into a shorter C-terminal fragment, termed DELE1-VS (50). This fragment is also able to activate the HRI kinase in the cytosol. The exact cleavage site by HtrA2 that yields DELE1-VS remains to be elucidated, but it is speculated to be between amino acids 456 to 466 (50). Surprisingly, although previous studies emphasized the importance of OMA1 for the cleavage of DELE1, OMA1 was dispensable in HEK293T cells in this study (46, 47, 50). Previous work used HeLa cells to study the DELE1–HRI pathway, thus these findings suggest that the protease cleaving DELE1 may be cell line–specific (46, 48). These results stress the importance of studying pathways in different cell models.

The fact that both the DELE1-S and DELE1-VS fragments are able to activate HRI suggests that the VS segment contains the minimal activating segment (50). Indeed, deletions studies showed that the last TPR region, absent in DELE1-VS, was not necessary for activating the ISR (47). Furthermore, full-length DELE1 can also accumulate in the cytosol and activate HRI (49) (Fig. 2). Upon low intracellular iron availability, DELE1’s import into the mitochondria is stalled and no longer degraded by LONP1. The TPR domains of DELE1 face the cytosol, allowing for their interaction with HRI and for ISR activation (49). This result further supports the idea that a TPR region is responsible for activating HRI.

The structure of the C-terminal domain of DELE1 (DELE1-S) was determined by cryo-EM (52). The structure consists of an oligomer formed by eight identical DELE1 fragments that form a symmetrical cylindrical shape, with two back-to-back tetramers. The oligomerization of the C-terminal domain of DELE1 is mediated by two interfaces (interface I and II). Interactions at interface I promote tetramer formation of the DELE1-S fragments and those at interface II aid with the dimerization of the two tetramers. Importantly, mutations that disrupt oligomerization also impaired the activation of the ISR, suggesting that oligomerization of DELE1 is needed to promote this response. However, it is also possible that the same interface is required for binding HRI, in which case the mutations would also disrupt HRI activation directly. Nonetheless, the oligomerization of DELE1 acts as another layer of regulation for the ISR pathway, fine-tuning the process to ensure it is only activated upon exposure to a stressor.

The next major question is to determine how the DELE1 interacts with and activates HRI. There are four kinases that can activate the ISR (HRI, PERK, PKR, and GNC) that all have similar kinase domains; however they each have unique N-terminal regions (52). To determine the portion of HRI responsible for DELE1-dependent activation, binding assays were performed using different deletions of HRI (52). The N-terminal region (aa 1–160) of HRI alone copurified with DELE1, implying that HRI is activated *via* this interaction. HRI kinase activation has been extensively studied in other contexts, and it is known to require dimerization and autophosphorylation at Thr485 in mouse HRI (Thr488 in human) (53, 54, 55). The current working model for how DELE1 facilitates this process suggests that DELE1 acts as a template for the activation of the HRI kinase, with the DELE1 oligomer promoting the dimerization and autophosphorylation of HRI (52). DELE1 binds HRI at the N-terminal domain, allowing for the C-terminal kinase domain to remain available for binding to eIF2α (52). This speculative mechanism of action would be similar to the kinase PKR, another kinase associated with the ISR (56). The structure of DELE1 bound to HRI could clarify this mechanism and reveal potential therapeutic targets.

### Working together: the UPR^mt^ and DELE1-HRI ISR

The DELE1-HRI ISR and the UPR^mt^ are two critical and distinct pathways that mitochondria use to protect themselves from stress. These pathways exhibit downstream synergy, as they both activate stress-response genes, such as ATF4 and CHOP, to help repair cellular damage. This suggests that there may be a common feature between the two pathways. In fact, when DELE1 is knocked-out in cells, the upregulation of UPR^mt^ genes is reduced (23). In contrast, depletion of ATF4 or ATF5 does not affect UPR^mt^ activation (23, 29). These findings highlight DELE1 as a potential common upstream factor for both pathways and suggest that the ISR is an essential precursor to UPR^mt^ activation (57). Taken together, these observations propose that when mitochondria are exposed to stress, the first quality control mechanism activated is the DELE1-HRI ISR. Upon activation, this response slows cytosolic translation, serving as an initial attempt to alleviate mitochondrial stress (57). As the stress persists, the DELE1–HRI axis stimulates the release of specific stress-response genes, helping with cellular repair. In parallel (perhaps as ROS levels increase due to stress), UPR^mt^ genes are upregulated, leading to increased expression of chaperones and proteases. These responses work together to help repair the damaged mitochondria compartment and maintain proteostasis. This proposed model in mammalian cells differs from the findings in *C. elegans*, where the ISR has been shown to be dispensable for UPR^mt^ activation (57). Further studies are needed to explore the overlap between the ISR and UPR^mt^ in mammalian models and to determine if the pathways are activated differentially under distinct stressors. Additionally, understanding why these pathways are interconnected in mammalian cells, but not in *C. elegans models,* could provide insights into how the mitochondrial stress responses evolved.

## Removal of damaged mitochondria *via* the endolysosomal pathway

**Figure 3:**
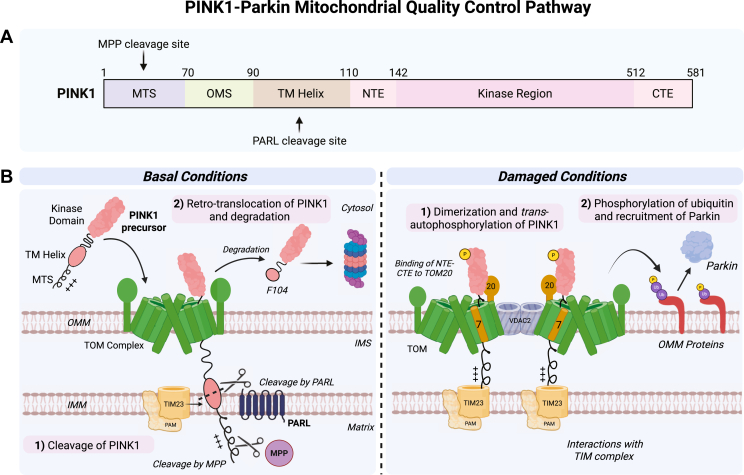
**The PINK1–Parkin Pathway under healthy and damaged conditions**. *A*, PINK1 consists of a mitochondrial targeting sequence, outer mitochondrial membrane signal, transmembrane helix, and an N-terminal extension and a C-terminal extension to the kinase domain. *B*, under healthy conditions, PINK1 is imported into mitochondria through the translocase of the outer membrane (TOM), translocase of the inner membrane (TIM23), and presequence-associated motor (PAM) complexes. Once inside, PINK1 is rapidly cleaved by mitochondrial processing peptidase between Ala28 and Tyr29. PGAM5-associated rhomboid-like protease then cleaves PINK1 between Ala103 and Phe104, triggering retro-translocation of PINK1 to the cytosol, where it is rapidly degraded by the proteasome. Under damaged conditions, PINK1 accumulates on damaged mitochondria by interacting with the TOM and TIM23 complexes. The TOM complexes are arranged in a symmetrical fashion around a central VDAC2 dimer. TOM7 and TOM20 play critical roles in stabilizing PINK1; TOM20 binds to the N-terminal extension–C-terminal extension of PINK1. Following accumulation, PINK1 becomes activated through dimerization and *trans*-autophosphorylation. Once active, PINK1 phosphorylates nearby ubiquitin present on outer mitochondrial membrane proteins, which in turn recruits and activates the E3 ubiquitin ligase Parkin to mediate mitophagy. PINK1, PTEN-induced kinase 1.

Under certain circumstances, the ISR and UPR^mt^ pathways may become insufficient to repair mitochondrial damage. This can occur when stressors, such as oxidative stress, mitochondrial membrane depolarization, or unfolded protein aggregates, become too severe for these pathways to mitigate (10). Under these circumstances, damaged mitochondria are degraded *via* the autophagy and endolysosomal system, a process that is generally known as mitophagy. In this context, the PINK1–Parkin pathway emerged as a key mechanism for the selective turnover of damaged mitochondria by mitophagy (Fig. 3). Notably, when the UPR^mt^ is not functional, the PINK1–Parkin pathway compensates, highlighting the interplay between these two pathways and suggesting that PINK1-Parkin acts as “last resort” to remove damaged mitochondria, although the context and availability of different effectors may affect the extent of each pathway (23). In the following sections, we will describe the PINK1–Parkin pathway, focusing on its biology and recent structural studies. We will then compare the PINK1-Parkin and HRI-dependent mitophagy responses and explore how these pathways may interact.

### PINK1–Parkin mitochondrial quality control pathway

The PINK1–Parkin mitochondrial quality control pathway is one of the most extensively studied mitophagy responses, since loss of function mutations in these proteins have been linked to early-onset Parkinson’s disease (58, 59). The first studies indicating these proteins worked together in the same pathway came from *Drosophila*, where it was shown that deletion of Parkin or PINK1 caused flight muscle degeneration and mitochondrial pathology (60, 61, 62). Since then, numerous studies have shown how the kinase PINK1 recruits and activates the E3 ubiquitin ligase Parkin to mediate mitochondrial turnover. Here, we will focus on key aspects about the pathway and recent advances, as the topic has been extensively reviewed elsewhere (12, 63).

PINK1 is a mitochondrial serine/threonine kinase that functions as a damage sensor by accumulating and becoming active only on damaged mitochondria (64). Under basal conditions, PINK1 is rapidly degraded and maintained at a low steady-state level. PINK1 is composed of an atypical MTS (residues 1–90), a transmembrane helix (residues 94–110), and a cytosolic domain containing the kinase activity (residues 110–581) (65). Like DELE1, PINK1 is imported into the mitochondrial matrix *via* the presequence import pathway under basal conditions (66, 67, 68) (Fig. 3). Once in the matrix, PINK1 is cleaved by mitochondrial processing peptidase between Ala28 and Tyr29 followed by cleavage of its transmembrane domain by the IMM protease PARL between Ala103 and Phe104 (66, 67, 69, 70). PINK1 is then retro-translocated to the cytosol, where it is degraded by the proteasome (71, 72, 73). In the cytosol, cleaved PINK1 is recognized by the E3 ubiquitin ligases UBR1, UBR2, or UBR4 and is subsequently degraded *via* the proteasome (71). The sorting and processing of PINK1 under basal conditions likely explains why its half-life is only approximately 30 min (74, 75). On the contrary, when mitochondria become depolarized, PINK1’s half-life increases from minutes to hours, resulting in the accumulation of PINK1 on damaged mitochondria and activation of its kinase activity (74). Exposure to stressors that disrupt the mitochondrial membrane potential, such as the protonophore carbonyl cyanide chlorophenylhydrazone, dual inhibition/knockdown of complex III/V in the electron transport chain, mtDNA mutations, or expression of the short mitochondrial ARF lead to PINK1 accumulation on damaged mitochondria through its interaction with the TOM and TIM complexes (64, 74, 76, 77, 78, 79). Once bound to the TOM complex, PINK1 becomes active by dimerizing and undergoing *trans*-autophosphorylation at Ser228 (80, 81, 82). This activation allows PINK1 to phosphorylate ubiquitin at Ser65 on nearby OMM substrates (83, 84). Phospho-ubiquitin (pUb) anchored on mitochondrial proteins then recruit Parkin to the damaged mitochondria through a high affinity interaction.

Parkin is a RING-between-RING E3 ubiquitin ligase made up of a regulatory N-terminal ubiquitin-like (Ubl) domain and a catalytic C-terminal domain (85). Upon binding pUb on damaged mitochondria, the Ubl dissociates, which allows PINK1 to phosphorylate it at Ser65 (78, 84, 86). Once phosphorylated, Parkin becomes catalytically active and ubiquitinates OMM substrates such as mitofusins and voltage-dependent anion channels (VDACs) (83, 86). The newly added ubiquitin on these substrates can also be further phosphorylated by PINK1, which can recruit additional Parkin molecules to the damaged mitochondria. This mechanism acts as a positive feedback loop that results in the formation of phosphorylated polyubiquitin chains on the mitochondrial surface (87). The polyubiquitin chains serve as signals to recruit autophagy receptors, such as optineurin, which facilitate the formation of an autophagosome (88). The autophagosome can then fuse with lysosomes, leading to the degradation of the damaged mitochondria through mitophagy (10, 88).

Although the PINK1–Parkin quality control pathway is often associated with mitophagy, there have been ongoing debates over the years about whether there are alternative pathways for damage removal activated by PINK1 and Parkin. One of the first studies to spark this debate was conducted in *Drosophila*, where researchers showed that the degradation of only a subset of mitochondrial proteins depended on PINK1 and Parkin, and that this subset only partially overlapped with proteins affected by the loss of ATG7, an enzyme required for autophagosome formation (89). This suggested that the removal of mitochondrial proteins through the PINK1–Parkin pathway may not be solely mediated by autophagy and that other autophagy-independent pathways might be involved. Indeed, it was later found that the PINK1–Parkin pathway can also facilitate damage removal through the formation of mitochondrial-derived vesicles (MDVs) in response to oxidative stress (90). MDVs bud off from the mitochondria independent of mitochondrial fission machinery and transport damaged cargo to the lysosomes for degradation (91). Unlike mitophagy, this mechanism salvages the mitochondria, removing only the damaged compartment. It is speculated that the PINK1–Parkin pathway will first initiate the formation of MDVs to remove damage and if the damage is too severe, and then mitophagy will take over. More work is needed to better under the mechanism behind the MDV pathway and its relationship to PINK1 and Parkin. These studies suggest that PINK1-Parkin can trigger various responses, depending on the severity, spread, and duration of the damage, ranging from MDV formation to gradual mitophagy of mitochondria undergoing fission (92, 93).

### Structural basis for the activation of PINK1 on the TOM and TIM complexes

For the purpose of this review, we will not describe in details the structures of Parkin and PINK1, which have been discussed by our group in 2023 (12). Instead, we will focus on recent structural advances regarding how PINK1 accumulates on damaged mitochondria. While it is well established that PINK1 accumulates on damaged mitochondria, the structural mechanism behind this process has been unclear, though some insights are available. Upon mitochondrial depolarization, PINK1 forms an 800 kDa complex with TOM, an import complex made of 7 subunits (94). In cells lacking the accessory subunit TOM7, PINK1 no longer accumulates on damaged mitochondria (95). In the absence of TOM7, PINK1 is degraded by the IMM protease OMA1 under depolarized conditions (96). Thus, TOM7 plays a critical role in stabilizing PINK1 by preventing its degradation. TOM20, the presequence receptor subunit of the TOM complex, also plays an essential role in stabilizing PINK1 under stress conditions. Disruption of the PINK1–TOM20 interaction results in the failure of PINK1 accumulation and activity (97, 98). Furthermore, specific regions in PINK1 are also important for its accumulation. Three negatively charged glutamic acid residues (Glu112, Glu113, and Glu117), located immediately after the transmembrane domain of PINK1, are required for its stabilization (96). In addition to these residues, a specific segment of PINK1’s MTS known as the outer mitochondrial membrane signal (OMS), spanning residues 70 to 90, is essential for PINK1's accumulation on the TOM complex (68). Deletion of the OMS results in PINK1 degradation by OMA1. Unlike the DELE1 response, where OMA1 is crucial for activating the pathway, OMA1 is not required for PINK1 activation and in fact only degrades PINK1 when these essential elements required for its stabilization are missing. In this context, it is critical to understand how PINK1 is stabilized on the TOM complex and how each of these factors are important for PINK1’s activity.

To address these questions, the structure of the human PINK1–TOM complex was solved using cryo-EM by the lab of David Komander (99). This structure reveals how human PINK1 accumulates on the TOM complex under conditions of mitochondrial depolarization. Previously, the structure of the cytosolic kinase domain of insect PINK1 had been solved by Komander’s group and our own, offering initial insights into the mechanisms of autophosphorylation and stabilization on damaged mitochondria (100, 101). The structure of the human PINK1 kinase domain closely resembles that of insect PINK1, with PINK1 forming a symmetric dimer that enables autophosphorylation in *trans* at Ser228 (99). In addition, the human structure revealed an intermolecular disulfide bond at cysteine 166, which appears to stabilize the dimer (99). PINK1 is unlikely to phosphorylate nearby substrates in this state, indicating that the observed structure represents a preactive conformation. For PINK1 to function as an active ubiquitin kinase, the dimer would need to dissociate, a process that would require reduction of the disulfide bond. Further structural studies are needed to capture this active state of human PINK1 and better understand how the dimer is able to dissociate under damaged conditions.

The cryo-EM structure also revealed that the symmetrical PINK1 dimer accumulates on top of two TOM core complexes, each composed of two copies of TOM40, TOM7, TOM6, TOM5, and TOM22, as well as one copy of TOM20. These TOM complexes are arranged in a strikingly symmetrical fashion around a central VDAC2 dimer (Fig. 3). VDACs are some of the most abundant proteins on the OMM and are major substrates of Parkin in mitophagy (102, 103). While VDAC1/3 are dispensable for Parkin-dependent mitophagy, knockdown of VDAC2 in VDAC1/3 KO fibroblasts reduces Parkin recruitment to depolarized mitochondria (104). However, it was not previously thought to contribute to PINK1 stabilization on depolarized mitochondria. This unexpected structural arrangement raises several important questions for further research, including whether VDAC2 is required for PINK1 stability or activity, and whether the TOM–VDAC interaction is also important for the import of other mitochondrial proteins in mammalian cells. However, we did not observe specific enrichment for VDAC2 in our affinity purification of PINK1, whereas TOM20 and all other core TOM subunits were present.

Another key feature observed in the structures of both human and insect PINK1 is the presence of both an N-terminal extension (NTE) and a C-terminal extension (CTE) to the kinase domain, which bind to each other on the opposite side of the kinase active site. Importantly, disruption of the NTE–CTE interaction prevents PINK1 from associating with the TOM complex, indicating that this interface is also critical for PINK1 stabilization (100, 105). Using affinity proteomics and AlphaFold modeling, our group found that TOM20 binds to the NTE-CTE region of PINK1, clarifying its importance for PINK1 activation (97). This direct interaction was observed in the structure of the human PINK1–TOM complex (99). Together, these findings demonstrate that PINK1 is anchored on the TOM complex and stabilized through key interactions with both TOM5 and TOM20. In this conformation, the N terminus of PINK1 is threaded into the TOM complex pore, where the C terminus of TOM7 interacts with PINK1 at the TOM40 exit in the IMS, while TOM7’s N terminus interacts with phosphatidylcholine, thus revealing a possible explanation for TOM7’s importance in PINK1 accumulation. These structural findings match *in vitro* reconstitution of the entire PINK1–TOM complex in budding yeast which confirmed that TOM20 and TOM7 play essential roles in PINK1’s activation (106). This study also showed that TOM70, a cochaperone that interacts with HSP90 and is involved in regulating precursor import, is important for PINK1 activity. Intriguingly, our lab saw no enrichment of TOM70 upon affinity purification of PINK1 and it is absent from the PINK1-TOM structure. Thus, while TOM70 may not be part of the PINK1–TOM complex, it could nonetheless play a critical role in the initial assembly of the complex. Future work is needed to determine the specific contributions of TOM70, TOM7, TOM20, and VDAC2 to PINK1 stabilization. Additionally, the region predicted to form a transmembrane helix in PINK1 does not adopt a helical structure in the resolved human PINK1 structure (99). Instead, it serves as a linker between the OMS and the NTE of PINK1, positioning the OMS within the TOM40 barrel, highlighting its importance for PINK1 stability.

Using affinity proteomics, the lab of Toshihiko Oka as well as our lab also found that the 800 kDa complex of PINK1 comprises not only the TOM but also the TIM23, TIM17, and TIM50 subunits of the TIM23 presequence import complex (97, 107). Knockdown of the TIM50 subunit, which bridges the TOM and TIM23 complexes, impaired PINK1’s ability to accumulate on damaged mitochondria and become active, suggesting that binding to the TIM23 complex is essential for PINK1 stabilization. While solving the human PINK1-TOM structure, Komander’s group also found crosslinks between PINK1 and the TIM23 complex (99). With all of these findings, a new model has emerged for how PINK1 accumulates and stabilizes on damaged mitochondria to recruit Parkin. Under basal conditions, PINK1 is imported through the TOM and TIM complexes, which places the transmembrane domain at the IMM so that PINK1 can get cleaved by PARL. This positioning results in the NTE of PINK1 being in the IMS, thus unable to bind the CTE and impeding the folding and activation of the kinase domain. However, when mitochondria become depolarized, the MTS of PINK1 interacts with the TIM23 complex but does not translocate into the matrix. This blockade allows enough time for the NTE-CTE module to fold in the cytosol and bind TOM20, which stabilizes PINK1 on the TOM complex. In this model of the damaged state, PINK1’s transmembrane domain would be positioned within the TOM complex and would protect the domain from cleavage. This arrangement would leave the kinase domain of PINK1 exposed in the cytosol, where it can then dimerize and enable autophosphorylation (Fig. 3). Komander further proposes that PINK1 dimerizes on two core TOM complexes and VDAC, but in order for PINK1 to be an active kinase, it needs to dissociate. They suggest that as ROS levels increase in response to mitochondrial damage, the PINK1 dimer and the TOM-VDAC array dissociate, allowing VDAC to become ubiquitinated and PINK1 to phosphorylate the ubiquitin, enabling Parkin recruitment. However, the precise sequence and regulation of these events remain to be fully elucidated.

All studies on the PINK1–TOM–TIM23 complex point toward the PINK1–TIM23 interaction being labile, with TIM23 subunits coeluting in substoichiometric amounts or needing crosslinking. This raises the question: what regulates PINK1’s interaction with the TIM23 complex? In a review from Richard Youle’s lab, it was proposed that the positively charged MTS of PINK1 interacts with an acidic patch in TIM17, a subunit of the TIM23 complex, which becomes accessible only under depolarization (63). In contrast, under basal conditions, the MTS is pulled through the TIM23 complex with the help of the presequence-associated motor (PAM) complex, which facilitates ATP-dependent translocation of proteins into the mitochondrial matrix. The model explains the importance of the three glutamic acid residues in PINK1. These residues are located at the onset of the NTE and thus may slow the import of PINK1 into the TOM complex, allowing time for proper folding of the NTE-CTE region. The model remains speculative and there are many questions that remain to be examined. It is unclear how the TIM23 complex changes conformation upon depolarization. It was recently suggested that ROMO1 (Mgr2 in yeast), which is part of the TIM23 complex, may regulate this process (108). ROMO1 was not found in our affinity proteomics screen (97), suggesting it may dissociate from the TIM23 complex upon depolarization.

The formation of the TOM–TIM23 supercomplex in yeast has been resolved using cryo-EM, employing an artificial construct with an MTS linked to a superfolder GFP that folds in the cytosol, effectively trapping the MTS on both import complexes and providing a snapshot of a transient import intermediate. (109). PINK1 is the first endogenous substrate capable of similarly linking the two channels together. It remains to be understood the interactions with the TIM but the most likely model is for PINK1 to form a dimer on a tetrameric TOM complex with VDAC2, with each MTS binding a TIM23 complex. Structural investigations will be crucial for gaining insights into the changes that occur in the import complexes under damaged conditions, specifically for the IMM.

### Crosstalks between PINK1/Parkin and the UPR^mt^

The PINK1–Parkin pathway can be activated not only by mitochondrial membrane depolarization but also through the accumulation of misfolded proteins. For example, when the deletion mutant ΔOTC is expressed in cells, PINK1 accumulates and triggers mitophagy (110). OTC is a mitochondrial matrix protein, and its partial deletion leads to misfolding and accumulation in the matrix (110). This mutant was initially found to activate the UPR^mt^ (19, 20), suggesting that the PINK1–Parkin pathway and UPR^mt^ share a common inducer. Interestingly, expression of ΔOTC does not affect the mitochondrial membrane potential, indicating that the PINK1–Parkin pathway can be activated even when the membrane potential remains intact (110). Another stressor that induces both UPR^mt^ and PINK1–Parkin pathway activation is exposure to GTPP, a compound that inhibits the mitochondrial chaperone TRAP1 and leads to protein misfolding (18). Similar to ΔOTC, exposure to GTPP results in the accumulation of PINK1 on damaged mitochondria, subsequently triggering Parkin recruitment, independent of membrane depolarization, which inhibits the mitochondrial chaperone TRAP1 and causes protein misfolding (18). To determine how proteostasis stress can activate PINK1/Parkin, a CRISPR/Cas9 screen was conducted to identify components that could stimulate mitophagy even when mitochondria remain polarized (111). This screen revealed that inactivation of the PAM complex induces mitophagy. Proteins entering *via* the presequence pathway are driven into the matrix through the ATP-dependent activity of the PAM complex. Therefore, disruption of the PAM complex impairs protein import, even in the absence of depolarization. Mitochondrial protein misfolding also reduces protein import by disrupting the TIM23–PAM complex, thus preventing PINK1 from being imported through the IMM. This prevents PINK1 from being cleaved by PARL, leading to its stabilization. This model suggests that the PAM complex acts as a sensor for the UPR^mt^, in addition to ROS. However, this model is inconsistent with the lack of PINK1 accumulation upon deletion of mtHsp70, an essential component of the PAM complex (96) or by the lack of PINK1 activation upon inhibition of TIM23 import with stendomycin (112). It is also important to note that unfolded proteins increase ROS levels in the mitochondria, which could potentially induce PINK1-Parkin MDV formation (90). Reducing agents also abrogate PINK1-Parkin mitophagy (113). Interestingly, we demonstrated that PINK1 can assemble into the 800 kDa complex with TOM and TIM23 under conditions of misfolded protein stress (97). Both accumulation paradigms likely implicate the same trigger, which could involve remodeling of disulfide bonds. In this context, it is intriguing to find that the structure of the PINK1–TOM–VDAC2 complex contains multiple disulfide bonds, not only between PINK1 protomers but also between VDAC2 protomers and within PINK1 (99), but whether these disulfide bonds are required for formation of the complex remains to be tested.

### Working together or in parallel? HRI and PINK1 kinases both drive mitophagy

Several mitophagy pathways coexist in cells with the PINK1/Parkin pathway (reviewed in (38, 39)). For instance, exposure to the iron chelator deferiprone induces mitophagy independently of Parkin/PINK1 (114). David Chan’s lab found that deferiprone-induced mitophagy is mediated by the DELE1–HRI pathway (115). Specifically, activation of the HRI kinase leads to the accumulation of phosphorylated eIF2α on the surface of damaged mitochondria, which signals the initiation of mitophagy (Fig. 2). However, there was no increase in phosphoSer65-ubiquitin levels, suggesting that PINK1 was not involved in the process, as it is the only known kinase that phosphorylates ubiquitin at Ser65 (116). Thus, Chan’s team concluded that the HRI and PINK1-Parkin mitophagy pathways operate in parallel. However, a recent publication from the Muqit lab challenges the idea that there is no interplay between the PINK1–Parkin and DELE1–HRI mitophagy pathways (117). The researchers performed a genetic siRNA screen to identify kinases that might regulate PINK1’s activity. Surprisingly, they found that knocking down HRI led to increased PINK1 stabilization and activity. Moreover, they demonstrated that knocking down upstream components of the HRI stress response, such as DELE1 and OMA1, also enhanced PINK1 stabilization. The effect appears to be largely transcriptional, as depolarization and HRI knockdown cause an increase in the *PINK1* mRNA. Nonetheless, it was still dependent on translation, as cycloheximide blocked PINK1 accumulation. This is consistent with previous observations by the group of Xuedong Liu, who found that glucose depletion, which reduces the ATP production in depolarized cells, or partial translation inhibition blocked the PINK1/Parkin pathway *via* inhibition of PINK1 *de novo* synthesis (118). Based on these findings, the Muqit group proposes a new model in which, under mitochondrial stress, OMA1 is activated and cleaves DELE1 into a smaller fragment, DELE1-S (46, 47). DELE1-S then binds to HRI in the cytosol, activating the kinase. Once active, HRI negatively regulates PINK1, while simultaneously activating genes such as ATF4, which help the cell repair itself (117). Thus, the DELE1–HRI pathway may also have a role in antagonizing the PINK1-Parkin response, perhaps to allow the cell to repair mitochondrial components before mitophagy induction.

Future studies are needed to understand the timing and threshold of activation of these different mitophagy responses, in different cellular context. This likely depends on the degree of damage and the abundance of their different effectors, and that emergent complex properties can emerge from relatively simple pathways that interact with each other. These emergent properties were modelled for the PINK1/Parkin pathway, which showed that Parkin recruitment requires a certain PINK1 threshold on mitochondria, and that the timing delay of Parkin recruitment is inversely proportional to the PINK1 concentration (87). A similar analysis could be performed to understand the activation dynamics of both pathways. Finally, targeting the HRI pathway could help regulate the PINK1–Parkin pathway when impaired, making it a key area for future research.

**Figure 4:**
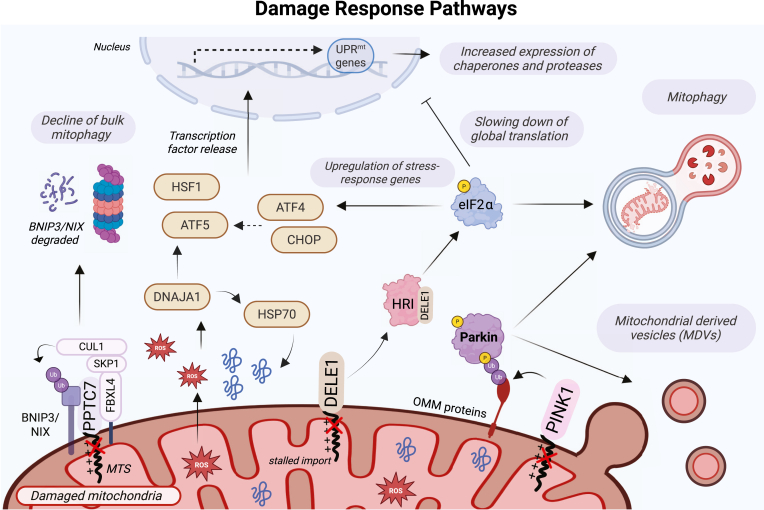
**Overview of the different stress responses that are activated by mitochondrial damage.** Under conditions of physiological stress, bulk mitophagy is slowed down by inhibiting BNIP3/NIX-mediated mitophagy. In this scenario, PPTC7 accumulates on the outer mitochondrial membrane, where it forms a complex with FBXL4 and NIX. This complex acts as a scaffold to promote the ubiquitination of BNIP3/NIX by the Skp1-Cul1-F-box–FBXL4 complex. Preventing the degradation of mitochondria helps control mitochondrial mass. In parallel, the DELE1-HRI integrated stress response reduces stress by slowing down translation and thus reducing mitochondrial biogenesis. DELE1 localizes to the cytosol, where it activates the kinase HRI, which then phosphorylates eIF2α. This will slow cytosolic translation, serving as an initial attempt to alleviate mitochondrial stress. If damage persists, specific genes will be upregulated, needed for cellular repair (ATF4 and CHOP). In parallel, the mitochondrial unfolded protein response (UPR^mt^) will be activated, triggering the upregulation of UPR^mt^ genes (HSF1, ATF5), which cause increased expression of mitochondrial chaperones and proteases. If damage becomes too severe and persists, the mitochondria will undergo mitophagy, primarily *via* the PINK1–Parkin pathway. Similar to DELE1 and PPTC7, PINK1 also accumulates on damaged mitochondria, where it helps recruit Parkin to initiate mitophagy. The PINK1–Parkin pathway can also remove damaged mitochondria through the formation of mitochondrial-derived vesicles and it is speculated they first remove damage through mitochondrial-derived vesicles before using mitophagy. The DELE1–HRI pathway can stimulate mitophagy as well, but the exact time and mechanism it does so remains unclear, perhaps it does so in parallel to PINK1-Parkin mitophagy. eIF, eukaryotic translation initiation factor; PINK1, PTEN-induced kinase 1.

It is worth highlighting that there are some key similarities between the PINK1–Parkin and DELE1–HRI pathways, namely how the kinases PINK1 and HRI become active (Fig. 4). Both PINK1 and HRI are serine/threonine kinases that require dimerization and autophosphorylation for activation. Dimerization of PINK1 is essential for the activation process and serves as a precursor to autophosphorylation (100, 119). PINK1 dimerization takes place directly on the TOM complex, because this is where it folds upon binding TOM20. Similarly, HRI also requires dimerization for efficient autophosphorylation (53, 54). Like PINK1, two HRI molecules come together, with one molecule phosphorylating the other. For HRI to become active, it must bind to the oligomeric form of DELE1 (52). Thus, the binding of HRI to DELE1 and the binding of PINK1 to the TOM complex share similar mechanisms. Comparing the activation mechanisms of these two kinases offers structural insights into mitochondrial damage responses and suggests that kinase domain stabilization and autophosphorylation act as gatekeepers to prevent premature activation of these pathways.

### Maintaining mitochondria beyond stress conditions, the BNIP3/Nix pathway

The PINK1–Parkin and DELE1–HRI pathways are key responses involved in stress-induced mitophagy. However, mitophagy is not only crucial under pathological conditions but can also be activated under steady-state conditions in cells (120). Basal mitophagy is essential for routine mitochondrial maintenance, and if impaired, it can lead to health complications such as accelerated cellular aging (121). One of the best-studied pathways implicated in basal mitophagy is the BNIP3/NIX pathway (122, 123). This pathway induces mitophagy through a receptor-mediated mechanism. NIX (also known as BNIP3L) and BNIP3 are receptors located on the OMM, where they bind to LC3 on the autophagosome, facilitating mitophagy (124, 125). Interestingly, dimerization of these receptors has been shown to more effectively trigger the recruitment of autophagic machinery *via* phosphorylation, in a manner reminiscent of PINK1 and DELE1/HRI (126, 127). The significance of this receptor-mediated pathway was first highlighted for its role in the development of reticulocytes (128, 129). Since this discovery, NIX-mediated mitophagy has also been associated with the development of neuronal cells, natural killer cells, and renal cells, where it contributes to the programmed degradation of mitochondria (130, 131, 132). Unlike the PINK1-Parkin and DELE1-HRI responses, which mitigate mitochondrial damage, this form of mitophagy supports cellular differentiation. Additionally, both NIX and BNIP3 are upregulated on mitochondria under hypoxic conditions, where they eliminate excess mitochondria to aid in cellular adaptation to low-oxygen environments (125). Thus, receptor-mediated mitophagy plays a critical role in controlling mitochondria mass, ensuring proper mitochondrial maintenance according to the cell’s needs (Fig. 4) (123).

The BNIP3/NIX pathway must be tightly regulated, as these receptors can actively eliminate mitochondria under programmed conditions. In 2023, three groups identified FBXL4 as a negative regulator of the BNIP3/NIX mitophagy pathway through CRISPR KO screens (133, 134, 135). FBXL4 is a mitochondrial-localized F-box protein, acting as a substrate receptor for the Cullin-1-RING ubiquitin ligase complex (135). Cullin-RING ligases are a large family of multisubunit E3 ligases that bind an adaptor subunit and an F-box protein, which confers substrate specificity (136). The binding of FBXL4 to Skp1 results in the formation of the Skp1-Cullin1-F-box (SCF) ubiquitin E3 ligase complex, which ubiquitinates BNIP3 and NIX for proteasomal degradation (134, 135). Loss-of function mutations of FBXL4 prevents the formation of the SCF complex, leading to excessive mitophagy and the development of mtDNA depletion syndrome 13, a degenerative disorder (135). FBXL4 therefore plays a crucial role in regulating BNIP3/NIX mitophagy.

Although these studies have provided a deeper mechanistic understanding of how the BNIP3/NIX pathway is regulated, the question remains as to when these receptors are degraded and when they are spared, depending on the specific needs of the cell. This question was answered with the discovery of PPTC7 as a master regulator of this mitophagy response (123, 137, 138). The knockout of PPTC7 indeed hyperactivates BNIP3/NIX-mediated mitophagy. PPTC7 is a phosphatase that is normally targeted to the mitochondrial matrix *via* the presequence import pathway. In a manner reminiscent of PINK1 or DELE1, PPTC7 accumulates on the OMM as a longer, unprocessed form under conditions that induce mitophagy such as iron depletion (Fig. 4). PPTC7 then forms a complex with FBXL4 and NIX and acts as a scaffold to induce the ubiquitination of BNIP3/NIX by the SCF–FBXL4 complex, independently of its phosphatase activity (137). Thus, PPTC7 acts as mitophagy sensor and antagonizes BNIP3/NIX to reduce basal mitophagy during the first instances of physiological stress (Fig. 4). For instance, PPTC7 is induced during starvation to prevent starvation-induced mitochondria loss and metabolic imbalances (123, 139). PPTC7 thus adds another layer of regulation to this pathway by integrating the homeostatic and physiological cues present in cells under steady state.

In summary, BNIP3/NIX-mediated mitophagy operates differently from the PINK1–Parkin and DELE1–HRI stress-induced mitophagy pathways. However, both the regulation of basal and stress-induced mitophagy mechanisms are essential for cellular health. Notably, the PINK1–Parkin pathway has also been shown to be active in skeletal and heart muscle cells under basal mitophagy conditions, but not in mammalian brain cells (140, 141, 142). The exact mechanism by which PINK1-Parkin becomes activated under basal state remains unclear. Additionally, whether PINK1–Parkin and BNIP3/NIX mitophagy pathways interact or overlap remains an area for further exploration, as this could have significant implications for therapeutic approaches.

## Relevance to human diseases and concluding remarks

The UPR^mt^, DELE1-HRI response, and PINK1–Parkin pathway all play crucial roles in maintaining mitochondrial health under conditions of stress. Disruption of any of these pathways has been linked to several human diseases, highlighting their importance in cellular function. For instance, the PINK1–Parkin pathway has been implicated in early-onset Parkinson’s disease, with loss-of-function recessive mutations in PINK1 or Parkin being the most common cause of the disease under 50 years old (12). Loss of Parkin or PINK1 likely cause excessive inflammation, as Parkin or PINK1 null mice display sensitivity to microbial pathogens (143, 144) and modulate inflammatory responses in response to mtDNA mutations or exhaustive exercise (145, 146). At the molecular level, many PINK1 mutations occur in the cytosolic domain and affect the kinase activity directly or the ability of PINK1 to become active. For example, the pathogenic C125G mutation affects the NTE of PINK1, and recent structural studies suggest that this mutation could disrupt proper protein folding and binding of PINK1 to the TOM complex, leading to loss of function (97, 105). These findings underscore the importance of structural studies to better understand PINK1's role in mitochondrial quality control. In fact, PINK1 has become a key therapeutic target, with companies like Mitokinin/AbbVie developing a drug that sensitizes PINK1 accumulation upon mitochondrial damage and increased its stability (147). However, the exact mechanism of this drug remains unclear, making further research into the PINK1–TOM–TIM complex critical for advancing therapeutic strategies. In addition, Biogen developed small molecule positive allosteric modulators for Parkin that increases the activity of the protein (148). Our lab and collaborators recently elucidated the mechanism of the Biogen activators, and discovered that they act like molecular glue to bridge Parkin with pUb at the site where phospho-Ubl binds, thus enabling the rescue of Parkinson’s mutation in the Ubl domain (149). The UPR^mt^ is also implicated in various diseases, including neurodegenerative disorders, cardiovascular diseases, and cancer (11, 150). In cancer, ATF5 and HSF1, which are both involved in the UPR^mt^, have been linked to oncogenesis (11, 150, 151, 152). ATF5 upregulates tumorigenic genes, promoting cancer cell proliferation and invasion, while HSF1 helps cancer cells evade immune surveillance (11, 151, 152). Therefore, targeting the UPR^mt^ may offer new therapeutic approaches for cancer treatment and can inhibitors of these factors can be used as alternatives for cancer treatments that are becoming unresponsive (153). The DELE1-HRI response has been associated with neurodegenerative diseases and heart disease. For instance, DELE1 plays a protective role in mitochondrial cardiomyopathy (154). In cardiomyocytes, DELE1 activation is essential for managing mitochondrial stress, and loss of this pathway increases cardiomyopathy (154). Lastly, a recent preprint from the group of Michael Lazarou shows that upregulation of the BNIP3/NIX mitophagy pathway with Roxadustat can compensate the loss of PINK1-mediated mitophagy in neurons (155). This emphasizes the importance of understanding how mitophagy pathways crosstalk and the need to correct dysregulation when they occur.

These examples highlight the importance of well-regulated mitochondrial quality control. When these pathways fail or are dysregulated, they can lead to serious health complications. Further research is needed to better understand the individual roles of each pathway and how they interact with one another. These insights will be crucial for developing targeted therapeutic strategies to address the various mechanisms implicated in human diseases.

## Conflict of interest

The authors declare that they have no conflicts of interest with the contents of this article.
